# Immuno(T)herapy for age‐related diseases

**DOI:** 10.15252/emmm.202216301

**Published:** 2022-11-14

**Authors:** Enrique Gabandé‐Rodríguez, Matilda Pfeiffer, María Mittelbrunn

**Affiliations:** ^1^ Departamento de Biología Molecular, Facultad de Ciencias (UAM), Centro de Biología Molecular ‘Severo Ochoa’ (CSIC‐UAM) Universidad Autónoma de Madrid Madrid Spain; ^2^ Instituto de Investigación Sanitaria del Hospital 12 de Octubre (i+12) Madrid Spain; ^3^ Consejo Superior de Investigaciones Científicas (CSIC), Centro de Biología Molecular ‘Severo Ochoa’ (CSIC‐UAM) Universidad Autónoma de Madrid Madrid Spain

## Abstract

During the last decade, the stimulation of T‐cell function by the blockage of immunosuppressive checkpoints has experienced an outstanding impact in the treatment of cancer. Development of the chimeric antigen receptor T‐cell technology has also emerged as a powerful alternative for patients suffering from oncological processes, especially those affected by hematological neoplasms. Recent evidence suggest that the use of immunotherapy could be extended to non‐oncological diseases and could be especially relevant for age‐associated disorders, opening exciting therapeutic options for a wide range of diseases of the elderly. Here we comment on the emergence of T‐cell‐based immunotherapies as feasible approaches that could revolutionize the future of *GeroScience*.

## Graphical abstract

In this Commentary, M. Mittelbrunn, E Gabandé‐Rodríguez and M. Pffeifer discuss T cell‐based immunotherapy for age‐related diseases.

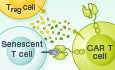

Mathematical projections estimate that the European population over 85 years old will triple by 2050. With the extension of life expectancy and the increased percentage of older individuals in the general population, understanding why aging results in progressively higher susceptibility to chronic morbidity, disability, and frailty has become a public health priority. To improve the quality of life, there is an urgent need to identify the molecular mechanisms common to the various diseases of aging and to find new therapeutic approaches to combat them simultaneously.

While the importance of chronic inflammation in the development of age‐associated diseases has been widely accepted, a causal contribution of immune cell dysfunction to inflammaging, systemic senescence, and aging has only recently been established (Desdín‐Micó *et al*, 2020; Yousefzadeh *et al*, 2021). Although both innate and adaptive immune cells undergo changes over time, these are especially evident in T cells. This susceptibility of T cells to age may be partly explained by the site of their generation, the thymus. The thymus is subjected to gradual involution starting with puberty and thus, its degeneration is considered one of the first manifestations of aging. Due to this thymic atrophy, there is a decline in the number of new T cells (recent thymic emigrates) that reach the periphery. T cells must then begin to perpetuate themselves by homeostatic proliferation of pre‐existing T‐cell clones. Due to this homeostatic proliferation and because they undergo clonal expansion upon antigen recognition, T cells are exposed to a great replicative pressure. During the successive rounds of divisions, T cells gradually lose their stemness and differentiate into terminally differentiated subsets, acquiring a senescent phenotype.

In our laboratory, we have generated a mouse model with prematurely aged T cells by forcing a severe mitochondrial failure due to a deletion of the mitochondrial transcription factor A in both CD4^+^ and CD8^+^ T cells. T cells from these mice display premature signs of aging, including the acquisition of a pro‐inflammatory and dysfunctional state, that reduces the ability to respond to acute infections and precipitates inflammaging. Strikingly, this was sufficient to trigger several age‐related diseases such as cognitive impairment, cardiovascular diseases, muscular and adipose tissue atrophy, metabolic alterations, and induced the accumulation of senescent cells in several tissues, suggesting that aged T cells can directly induce organismal aging and multimorbidity (Desdín‐Micó *et al*, 2020). Accordingly, mimicking immune system aging through the induction of DNA damage in the whole immune system induced senescence in multiple tissues (Yousefzadeh *et al*, 2021).

These findings suggest that immune cells, with T cells standing out, could directly act as inducers of systemic aging. However, how aged T cells contribute to the appearance of age‐related diseases is still unclear as the T‐cell compartment experiments profound, but still poorly characterized, changes during aging. These changes include the loss of the naïve T‐cell population and the accumulation of terminally differentiated memory T cells, some of them with a senescence, exhausted, or “natural killer” phenotype. In the last years, thanks to the application of single‐cell RNA sequencing techniques, the phenotypic and functional complexity of the different subpopulations of T cells that accumulate in aged tissues is beginning to be unraveled (Elyahu *et al*, 2019; Mogilenko *et al*, 2021). Both senescent and exhausted T cells display certain molecular hallmarks of aging, like mitochondrial decline and epigenetic remodeling (Carrasco *et al*, 2022). Exhausted T cells represent a non‐responsive subset characterized by a defective production of cytokines, which could be considered a protective response to avoid tissue damage. On the other hand, senescent T cells acquire extremely differentiated phenotypes, secreting high amounts of pro‐inflammatory and cytotoxic molecules and sometimes even acquiring innate immune cells characteristics which make them self‐aggressive and pathogenic. Supporting the pathogenic potential of senescent T cells in age‐related diseases, pioneer studies have linked the presence of senescent T cells to different age‐related pathologies. For instance, recent experiments have found increased numbers of clonally expanded CD8^+^ T cells with some features of senescence in the brains of patients with Alzheimer's disease (AD; Gate *et al*, 2020). These cells express TCRs that recognize antigens of the Epstein‐Barr virus and their presence has been inversely correlated with cognitive capacity. Additionally, it has been demonstrated that the age‐dependent decline of the regenerative potential of the nervous system is promoted by the pro‐inflammatory action of CXCR5^+^CD8^+^ T cells that are recruited to the spinal cord via CXCL13 (Zhou *et al*, 2022). In addition, T cells with senescent characteristics correlate with cardiovascular events. Altogether, these works suggest that aged T cells could directly induce organismal aging in different ways (Carrasco *et al*, 2022).

In the last decade, the development of immunotherapy has revolutionized the oncology field thanks to the use of *chimeric antigen receptor T cells* (CAR‐T cells) or the use of immune checkpoint inhibitors to reinvigorate T‐cell responses. In fact, Drs. James Allison and Tasuku Honjo saw recognized their groundbreaking findings on *immune checkpoint inhibitors* by the scientific community when they received the 2018 Nobel Prize in physiology or medicine. These innovative findings have supposed a spectacular advance in the treatment of some still incurable malignancies and have certainly opened a new era in the treatment of cancer. These strategies mainly target inhibitory molecules expressed by exhausted T cells, such as a programmed cell death‐1 (PD‐1) or cytotoxic T‐lymphocyte‐associated antigen 4. As recent discoveries suggest that T cells play a prominent role in different age‐related diseases, the use of these molecules has been proposed as a potential way to reinvigorate exhausted T cells during aging. First studies have tested the potential of anti‐PD‐1 blocking antibodies in mice with AD showing beneficial effects. In these experiments, PD‐1 blockade induced interferon gamma (IFNγ) T‐cell responses, promoting the recruitment of peripheral monocytes to the brain that ameliorated the pathology, most likely by decreasing Aβ plaque or Tau burden (Baruch *et al*, 2016). These works suggest that restoring exhausted T‐cell function could influence the entry and activity of other immune cells into the brain resulting in a beneficial effect in mouse models of neurodegenerative diseases. However, recent studies demonstrate that the use of immune checkpoint inhibitors aiming to reinvigorate exhausted T cells for cancer treatment was associated with a threefold higher risk of developing atherosclerotic cardiovascular events, including myocardial infarction, coronary revascularization, and ischemic stroke (Drobni *et al*, 2020). To avoid T‐cell overreaction and tissue damage during aging, it would be interesting to design the opposed strategy: to exhaust or eliminate senescent T cells instead of re‐activating exhausted cells (Fig 1). In this regard, different therapeutic strategies appear as options for this purpose: the depletion of senescent T cells using senolytic drugs, CAR‐T cells, or vaccines. Additionally, boosting T regulatory cell (T regs) function could be a plausible option to exhaust T cells.

**Figure 1:**
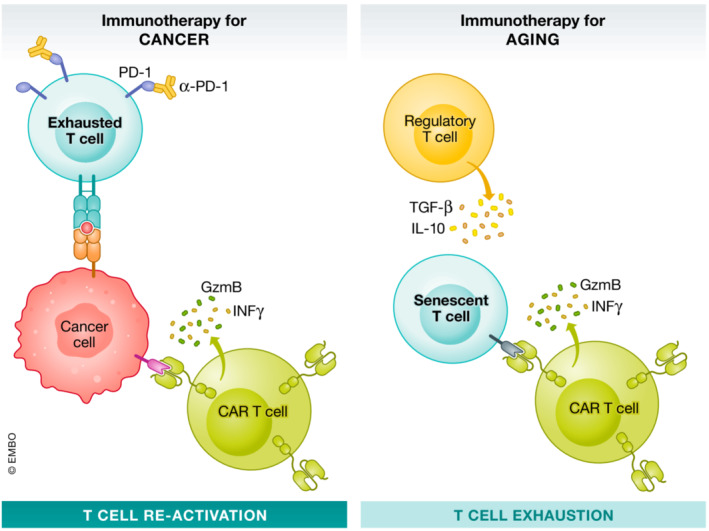
Immunotherapy strategies for the treatment of cancer and age‐related diseases Classically, immunotherapy strategies using immune checkpoint inhibitors, such as PD‐1 blocking antibodies, have focused on reinvigorating exhausted T cells to enhance the immune response against cancer cells. In addition, the generation of CAR‐T cells against cancer cell specific epitopes has served to eliminate malignant cells, especially in hematological neoplasms. During aging, the appearance of senescent T cells promotes the secretion of pro‐inflammatory and cytotoxic molecules contributing to inflammaging. In this context, strategies such as CAR‐T cells against senescent T‐cell‐specific antigens constitute promising strategies to target these cells. Additionally, boosting T reg functions might promote the “exhaustion” of senescent T cells, thus inhibiting their pathological properties.

Development of the *CAR‐T cell technology* has opened a new and very specific alternative to directly target pathological cells. Besides the common use of CAR T‐cells for hematologic cancers, senolytic CAR T‐cells directed against a senescence‐specific surface antigen have recently been developed. Thus, in mice with cardiac fibrosis, adoptive transfer of T cells expressing a CAR against the fibroblast activation protein effectively reduced fibrosis and restored cardiac function after injury (Aghajanian *et al*, 2019). In a recent, very exciting paper, Amor *et al* (2020) identified the urokinase‐type plasminogen activator receptor (uPAR) as a cell‐surface protein that is broadly induced during senescence and generated mouse uPAR‐specific CAR T‐cells. This strategy efficiently removed senescent cells in mice with liver fibrosis. A major caveat of this technology is that the clinical production of CAR‐T cells is still expensive and time‐consuming, and the generated CAR‐T cells usually do not last for long once injected back into the recipient. To overcome this, Rurik *et al* generated CAR‐T cells directly *in vivo* and used them to treat cardiac fibrosis in mice after cardiac injury. To do so, they injected CD5‐targeted lipid nanoparticles carrying a modified mRNA. The *in vivo* generated CAR‐T cells exerted anti‐fibrotic properties and restored cardiac function in mice, holding promising therapeutic potential in a wide range of diseases progressing with fibrosis (Rurik *et al*, 2022). These examples confirm that the generation of CAR‐T cells to eliminate senescent or pathogenic cells is a feasible strategy to ameliorate age‐related diseases. An alternative application with widespread beneficial effects would be the elimination of senescent T cells subsets by specific CAR‐T cells. However, the viability of this strategy depends on the identification of specific cell surface antigens exclusively expressed by senescent T cell subsets. A potential candidate could be CD153, which has been identified as a senescent T‐cell marker in the visceral adipose tissue of mice fed on a high‐fat diet (Yoshida *et al*, 2020). Immunization with a CD153 peptide sequence vaccine eliminated senescent T cells in the adipose tissue and improved glucose tolerance and insulin sensitivity in these mice suggesting that *vaccination* could be a therapeutic option to treat age‐related diseases.

Another potential approach to treat age‐related diseases could be the *induction of T regs*, which harbor immunosuppressive functions and control the activation of pathogenic T‐cell subsets. Boosting T reg responses has been largely used to prevent or delay a wide range of inflammatory and autoimmune diseases. Different strategies to induce T regs such as the administration of low doses of IL‐2 or the engineering of CD4^+^ T cells to stably re‐express FOXP3, the master transcription factor for T regs, have been used. These “induced” T regs cells are immunosuppressive and confer protection against inflammatory diseases but their potential to treat age‐related diseases is still to be addressed. As T regs suppress immune response, future studies should confirm whether T regs are able to transform senescent T cells into a non‐responsive or exhausted phenotype.

Based on the emerging role of T cells as accelerators of inflammaging, it seems clear that modulation of the senescent and exhaustion T‐cell programs could open immense opportunities, not only in anti‐tumor therapies, but also in preventing age‐related diseases. Gaining a more comprehensive understanding of the various T‐cell intrinsic and extrinsic stimuli that instruct T‐cell differentiation toward a dysfunctional state during aging is essential for designing effective strategies to promote healthy aging. We here introduce the idea of exhausting senescent T cells in age‐related diseases as a promising strategy to improve our resilience to age‐related disorders. Classically, PD‐1 has been considered a T‐cell exhaustion marker. However, a subset of CD8^+^ PD1^+^ T cells that secrete high amounts of granzyme K (GzmK), a factor that stimulates senescence in other cells such as fibroblasts, has recently been identified. This supports that different age‐associated T‐cell subsets accumulated in old tissues can promote tissue damage. Of note, the presence of this CD8^+^GzmK^+^PD‐1^+^ population correlates with markers of inflammaging such as IL‐6, IL‐8, and TNFα in aged humans (Mogilenko *et al*, 2021). These findings exemplify the importance of a better characterization of the different subsets of age‐associated T cells, uncovering additional markers that are exclusively expressed by exhausted or senescent T cells to design strategies to specifically target them.

During the last decade, research has witnessed the revolution of immunotherapy as a reality for cancer treatment. Very recent evidence suggest that T cells play a prominent role in the development of some age‐related diseases as relevant as myocardial infarction, atherosclerosis, neurodegeneration, or ischemic stroke. Thus, the use of immunotherapy to specifically target some pathogenic subsets of T cells now appears as a promising therapeutic option to simultaneously intervene in the course of different diseases whose incidence will exponentially grow in the next years.

## Author contributions

**Enrique Gabandé‐Rodríguez:** Conceptualization; writing – original draft; writing – review and editing. **Matilda Pfeiffer:** Conceptualization; writing – original draft; writing – review and editing. **María Mittelbrunn:** Conceptualization; funding acquisition; writing – original draft; writing – review and editing.

## Disclosure and competing interests statement

The authors declare that they have no conflict of interest.

## References

[emmm202216301-bib-0001] Aghajanian H , Kimura T , Rurik JG , Hancock AS , Leibowitz MS , Li L , Scholler J , Monslow J , Lo A , Han W *et al* (2019) Targeting cardiac fibrosis with engineered T cells. Nature 573: 430–433 31511695 10.1038/s41586-019-1546-z PMC6752964

[emmm202216301-bib-0002] Amor C , Feucht J , Leibold J , Ho YJ , Zhu C , Alonso‐Curbelo D , Mansilla‐Soto J , Boyer JA , Li X , Giavridis T *et al* (2020) Senolytic CAR T cells reverse senescence‐associated pathologies. Nature 583: 127–132 32555459 10.1038/s41586-020-2403-9 PMC7583560

[emmm202216301-bib-0003] Baruch K , Deczkowska A , Rosenzweig N , Tsitsou‐Kampeli A , Sharif AM , Matcovitch‐Natan O , Kertser A , David E , Amit I , Schwartz M (2016) PD‐1 immune checkpoint blockade reduces pathology and improves memory in mouse models of Alzheimer's disease. Nat Med 22: 135–137 26779813 10.1038/nm.4022

[emmm202216301-bib-0004] Carrasco E , Gómez de Las Heras MM , Gabandé‐Rodríguez E , Desdín‐Micó G , Aranda JF , Mittelbrunn M (2022) The role of T cells in age‐related diseases. Nat Rev Immunol 22: 97–111 34099898 10.1038/s41577-021-00557-4

[emmm202216301-bib-0005] Desdín‐Micó G , Soto‐Heredero G , Aranda JF , Oller J , Carrasco E , Gabandé‐Rodríguez E , Blanco EM , Alfranca A , Cussó L , Desco M *et al* (2020) T cells with dysfunctional mitochondria induce multimorbidity and premature senescence. Science 368: 1371–1376 32439659 10.1126/science.aax0860 PMC7616968

[emmm202216301-bib-0006] Drobni ZD , Alvi RM , Taron J , Zafar A , Murphy SP , Rambarat PK , Mosarla RC , Lee C , Zlotoff DA , Raghu VK *et al* (2020) Association between immune checkpoint inhibitors with cardiovascular events and atherosclerotic plaque. Circulation 142: 2299–2311 33003973 10.1161/CIRCULATIONAHA.120.049981 PMC7736526

[emmm202216301-bib-0007] Elyahu Y , Hekselman I , Eizenberg‐Magar I , Berner O , Strominger I , Schiller M , Mittal K , Nemirovsky A , Eremenko E , Vital A *et al* (2019) Aging promotes reorganization of the CD4 T cell landscape toward extreme regulatory and effector phenotypes. Sci Adv 5: eaaw8330 31457092 10.1126/sciadv.aaw8330 PMC6703865

[emmm202216301-bib-0008] Gate D , Saligrama N , Leventhal O , Yang AC , Unger MS , Middeldorp J , Chen K , Lehallier B , Channappa D , De Los Santos MB *et al* (2020) Clonally expanded CD8 T cells patrol the cerebrospinal fluid in Alzheimer's disease. Nature 577: 399–404 31915375 10.1038/s41586-019-1895-7 PMC7445078

[emmm202216301-bib-0009] Mogilenko DA , Shpynov O , Andhey PS , Arthur L , Swain A , Esaulova E , Brioschi S , Shchukina I , Kerndl M , Bambouskova M *et al* (2021) Comprehensive profiling of an aging immune system reveals clonal GZMK(+) CD8(+) T cells as conserved Hallmark of Inflammaging. Immunity 54: 99–115 33271118 10.1016/j.immuni.2020.11.005

[emmm202216301-bib-0010] Rurik JG , Tombácz I , Yadegari A , Méndez Fernández PO , Shewale SV , Li L , Kimura T , Soliman OY , Papp TE , Tam YK *et al* (2022) CAR T cells produced *in vivo* to treat cardiac injury. Science 375: 91–96 34990237 10.1126/science.abm0594 PMC9983611

[emmm202216301-bib-0011] Yoshida S , Nakagami H , Hayashi H , Ikeda Y , Sun J , Tenma A , Tomioka H , Kawano T , Shimamura M , Morishita R *et al* (2020) The CD153 vaccine is a senotherapeutic option for preventing the accumulation of senescent T cells in mice. Nat Commun 11: 2482 32424156 10.1038/s41467-020-16347-w PMC7235045

[emmm202216301-bib-0012] Yousefzadeh MJ , Flores RR , Zhu Y , Schmiechen ZC , Brooks RW , Trussoni CE , Cui Y , Angelini L , Lee KA , McGowan SJ *et al* (2021) An aged immune system drives senescence and ageing of solid organs. Nature 594: 100–105 33981041 10.1038/s41586-021-03547-7 PMC8684299

[emmm202216301-bib-0013] Zhou L , Kong G , Palmisano I , Cencioni MT , Danzi M , De Virgiliis F , Chadwick JS , Crawford G , Yu Z , De Winter F *et al* (2022) Reversible CD8 T cell‐neuron cross‐talk causes aging‐ dependent neuronal regenerative decline. Science 376: eabd5926 35549409 10.1126/science.abd5926

